# Host Immune Responses to Salivary Components - A Critical Facet of Tick-Host Interactions

**DOI:** 10.3389/fcimb.2022.809052

**Published:** 2022-03-16

**Authors:** Abid Ali, Ismail Zeb, Abdulaziz Alouffi, Hafsa Zahid, Mashal M. Almutairi, Fahdah Ayed Alshammari, Mohammed Alrouji, Carlos Termignoni, Itabajara da Silva Vaz, Tetsuya Tanaka

**Affiliations:** ^1^ Department of Zoology, Abdul Wali Khan University Mardan, Mardan, Pakistan; ^2^ King Abdulaziz City for Science and Technology, Riyadh, Saudi Arabia; ^3^ Department of Pharmacology and Toxicology, College of Pharmacy, King Saud University, Riyadh, Saudi Arabia; ^4^ College of Sciences and Literature Microbiology, Nothern Border University, Rafha, Saudi Arabia; ^5^ College of Applied Medical Sciences, Shaqra University, Shaqra, Saudi Arabia; ^6^ Centro de Biotecnologia, Universidade Federal do Rio Grande do Sul, Porto Alegre, Brazil; ^7^ Laboratory of Infectious Diseases, Joint Faculty of Veterinary Medicine, Kagoshima University, Kagoshima, Japan

**Keywords:** tick–host, crosstalk, salivary molecules, immune response, evasion mechanism

## Abstract

Tick sialome is comprised of a rich cocktail of bioactive molecules that function as a tool to disarm host immunity, assist blood-feeding, and play a vibrant role in pathogen transmission. The adaptation of the tick’s blood-feeding behavior has lead to the evolution of bioactive molecules in its saliva to assist them to overwhelm hosts’ defense mechanisms. During a blood meal, a tick secretes different salivary molecules including vasodilators, platelet aggregation inhibitors, anticoagulants, anti-inflammatory proteins, and inhibitors of complement activation; the salivary repertoire changes to meet various needs such as tick attachment, feeding, and modulation or impairment of the local dynamic and vigorous host responses. For instance, the tick’s salivary immunomodulatory and cement proteins facilitate the tick’s attachment to the host to enhance prolonged blood-feeding and to modulate the host’s innate and adaptive immune responses. Recent advances implemented in the field of “omics” have substantially assisted our understanding of host immune modulation and immune inhibition against the molecular dynamics of tick salivary molecules in a crosstalk between the tick–host interface. A deep understanding of the tick salivary molecules, their substantial roles in multifactorial immunological cascades, variations in secretion, and host immune responses against these molecules is necessary to control these parasites. In this article, we reviewed updated knowledge about the molecular mechanisms underlying host responses to diverse elements in tick saliva throughout tick invasion, as well as host defense strategies. In conclusion, understanding the mechanisms involved in the complex interactions between the tick salivary components and host responses is essential to decipher the host defense mechanisms against the tick evasion strategies at tick-host interface which is promising in the development of effective anti-tick vaccines and drug therapeutics.

## Introduction

The natural persistence and vectorial capabilities of ticks have been assisted by the evolution of sophisticated blood-feeding mechanisms and remarkable salivary molecules that come to play in the invasion of the vertebrate host immune responses (Wikel, 1982; Ribeiro, 1995; Wikel, 1996). Ixodid ticks are particularly different from Argasidae in the duration of their attachment to the host; they feed for several days depending on the life stage, as compared to soft ticks, which repeatedly feed for short periods of time ranging from minutes to hours (Oliver, 1989; Sonenshine, 1991). Due to the prolonged feeding, hard ticks have developed myriad strategies; they create a feeding cavity by piercing the host skin through the chelicerae that induce a blood pool in the dermis, where ticks inoculate saliva to facilitate blood-feeding, and remain attached to the host (Francischetti et al., 2009; Šimo et al., 2017; Boulanger and Wikel, 2021; Pham et al., 2021; Tirloni et al., 2021; Ali et al., 2022). These pharmacologically active molecules maintain the blood-feeding cavity and suppress the host defense mechanisms at the bite site (Nuttall and Labuda, 2004; Francischetti et al., 2009; Ali et al., 2015a; Martins et al., 2020). Consequently, essential tick salivary molecules are secreted into the bite site to deploy multiple proteolytic pathways that influence the host hemostatic and immunological responses against tick bite (Xu et al., 2016; Kotál et al., 2019; Zhou et al., 2020). These molecules possess a remarkable binding affinity toward host cytokines, eicosanoids, chemokines, growth factors, and other biological molecules (Andrade et al., 2005; Francischetti et al., 2009; Kazimírová and Stibraniova, 2013); inhibit the migration of neutrophils, leukocytes, and macrophages (MPs), and inactivate dendritic cells (DCs), mast cells (MCs), lymphocytes, eosinophils, keratinocytes, and endothelial cells (Kotsyfakis et al., 2006; Sá-Nunes et al., 2007; Bullard et al., 2019; Coutinho et al., 2020). As a result, the healing of the host’s wound is delayed by degrading pain-inducing molecular signals, host hemostasis, blood clotting, and immune responses (innate and adaptive) are disrupted at the site of the tick bite (Radulović et al., 2014). Thereafter, the tick can initiate a blood meal.

During a blood meal, ticks constantly secrete salivary molecules into the blood-feeding cavity to modulate the rigorous actions of the recruited host immune cells to avoid rejection by the host (Wikel, 1982; Wikel, 1985; Ribeiro, 1987; Kaufman, 1989; Ribeiro, 1989; Ramachandra and Wikel, 1992). Tick saliva has been recognized as being comprised of a large array of molecules, exerting potent immunosuppression by targeting multiple elements in the immune system of their respective hosts (Ribeiro, 1995). Several reviews have documented the specific roles of individual tick salivary molecules in the induction of host immunomodulation and/or inhibition, vasodilation, coagulation, platelet aggregation, and inflammatory responses (Šimo et al., 2017; Parizi et al., 2018; Bullard et al., 2019; Mans, 2020). However, a major debate remained undiscussed regarding the molecular mechanisms involved in the interaction of the tick–host interface. This review focuses on updated knowledge about the molecular mechanisms of various constituents of the host immune system and their associated proteolytic activities triggered by tick sialome. The growing knowledge of the molecular mechanisms has a critical role in the understanding of tick saliva in assisting tick feeding (Hovius et al., 2008a; Kotál et al., 2015; Šimo et al., 2017; Nuttall, 2019).

### Tick–Host Interface: Host Immune Components

The skin of the vertebrate host comprises a complex network of cellular interactions and exponentially relies on innate and adaptive immunity (Wikel, 2018; Boulanger and Wikel, 2021). In the early stage of tick feeding, immunosuppressive compounds are released in the tick saliva that encourages the formation of an effective feeding lesion (Ribeiro, 1987; Ribeiro, 1989; Nuttall et al., 2006; Francischetti et al., 2009). Ticks acquire blood meal by inserting their specialized mouthparts through the host skin. Fast feeding argasid deeply penetrates mouthparts to the host skin and feeds rapidly as compared to slow feeding ixodid ticks that can penetrate their mouthparts to the host skin superficially or deeply depending on tick species (Sonenshine, 1991). The inserted chelicerae extend and lacerate the epidermis, which is then followed by the insertion of the hypostome into the dermis (Richter et al., 2013). The tick-derived salivary complex cement cone proteins are secreted to facilitate tick attachment; they play a vital part in blood-sucking, sealing the feeding lesion, and immunomodulation thus enabling strong attachment mainly in hard ticks (Hollmann et al., 2018; Suppan et al., 2018). Cement cone proteins are of two types: a primary “core” (secreted early, within half an hour after attachment) and a secondary “cortex” cement (secreted for various days, having a graduated toughening procedure) (Bullard, 2016). Cement cone proteins fill any gap between the inserted mouthparts and non-intact host skin by using the polymerization of glycine-rich proteins secreted during early attachment (Bullard et al., 2019; Villar et al., 2020). Sealing improves blood-feeding and prevents fluid loss (Suppan et al., 2018). The secretion of cement cone proteins is copious in metastriate ticks (*Dermacentor* or *Rhipicephalus* genera) with short mouthparts as compared to prostriate ticks (genus *Ixodes*) with larger mouthparts (Ribeiro and Mans, 2020; Tahir et al., 2020). Successful attachment enables tick to conceal the scaffold of a combination of proteins, peptides, and non-peptide molecules that subsequently leads to the modulation of hemostasis, inflammation, wound healing, and the innate and adaptive immune responses of the host (Wikel et al., 1994; Reck et al., 2009; Wikel, 2018). During this course, ticks slowly start blood-feeding and ingest a small quantity during 4 to 5 days followed by a fast intake of blood meal in about 24 hours (Akov, 1982). Except for some *Ixodes* species, several tick species complete the final stage of their life cycle by mating on their host and subsequently take a large amount of blood in approximately 7 to 14 days before detachment and drop-off from the host (Kaufman, 2007; Anderson and Magnarelli, 2008).

A rapid cascade of events happens prior to and during tick blood-feeding (Tirloni et al., 2017). During this course, the salivary glands (SGs) yield and secrete a plethora of molecules in non-uniform time-dependent patterns (Kim et al., 2020), either to target-specific or a pleiotropic host’s molecules at the bite site, which initiates a set of mechanisms for disrupting host immune responses (Ren et al., 2019). This results in interference with several complement mediators—including complement cascade components, eicosanoids, chemokines, cytokines, growth factors, cell-signaling molecules, and antibodies (Aounallah et al., 2020) —proteolytic pathways, in particular pro-coagulants (thrombin, coagulation factors), pro-inflammatory enzymes (cathepsins S, C, B, L, and G, chymase, kallikrein, neutrophil elastase, proteinase 3, and tryptase), and complement pathway enzymes (component 2 and factors B, C, and D) (Tirloni et al., 2014; Kim et al., 2015a; Jmel et al., 2021). Meanwhile in this molecular tick–host crosstalk at the tick bite site, the dynamic responses of host hemostasis (first-line host innate immune response comprising platelet aggregation, coagulation, and vasoconstriction) are initiated against tick salivary molecules (Kim et al., 2020). This ultimately influences the composition and secretion of tick salivary components to meet the diverse requirements of tick attachment and moderate feeding, and to counter the dynamic responses generated by the host (Nuttall, 2019; Bonnet and Pollet, 2021; Boulanger and Wikel, 2021). Enhanced innate immune responses of the host against the rigorous activities of tick salivary molecules also activate the host inflammatory responses and complement cascade to counter block the tick salivary molecules (Preston et al., 2013; Kotál et al., 2015). Due to prolonged blood-intake and repetitive exposure of the host to ticks, the acquired immunity activates B lymphocytes and T lymphocytes (Olds et al., 2016; Šimo et al., 2017; Tabor et al., 2017). Ultimately, the tick–host interface leads to an arms race mechanism, where ticks try to evade the host skin and diminish the provoked immune system. In turn, the host develops early defense mechanisms against ticks (Kotál et al., 2015). The prolonged attachment and blood-feeding mechanisms of ticks are strengthened by drastic changes in the differential expression of tick salivary proteins in the repertoire of secreted saliva (Fontaine et al., 2011). During this endeavor, the host immune system takes time to recognize and counter the early secreted tick saliva–derived proteins and dynamics in its composition as a result of the hijacking of the host’s immune system (Tirloni et al., 2014; Mans, 2019). During blood-feeding, several proteins in the salivary repertoire are differentially expressed and enhances the hemoglobin digestion, heme transportation, blood coagulation, fibrinolysis, detoxification, and oxidative stress (Dickinson and Forman, 2002; Horn et al., 2009; Lara et al., 2015). Fundamentally understanding the differential expression provides significant incentives for determining the affinity and concentrations of tick salivary molecules against host components throughout the feeding period, which may be potentially useful for exploring tick-induced immunomodulation and the host’s enhanced defense mechanisms. Glycine-rich proteins present in the cement components are essential at tick feeding sites, since the RNAi-based gene knockdown induces reddening and bleeding around the mouthparts, interfering with the blood meal (Bullard et al., 2019). However, host-induced differences in the secretion of tick cement components during feeding among different tick species are mostly unexplored (Maruyama et al., 2010).

### Tick-Derived Salivary Inhibitors Modulating Host Hemostasis

The fate of the host’s innate immunity has been observed to be differentially modulated or inhibited by a set of tick salivary protease inhibitors (PIs) (Martins et al., 2020). A particularly notable role of PIs has been detected in tick–host crosstalk, as the host defense mechanisms are highly regulated by specific endogenous inhibitors subsequently inhibited by tick-secreted PIs (Martins et al., 2020; Jmel et al., 2021). The salivary PIs of several ticks have been characterized and found to be differentially expressed throughout the blood-feeding process, inducing local immunosuppression by inhibiting platelet aggregation or blocking cascade elements of intrinsic, extrinsic, and common pathways of blood coagulation, tumor development, and angiogenic factors (Chmelař et al., 2017; Costa et al., 2021). The host hemostatic mechanisms are not directly hindered by the tick salivary molecules; instead, the active sites, exocites, and receptors of regulatory factors or components involved in driving mechanisms such as thrombin, plasmin, factor V (fV), factor Xa (fXa), kallikrein, and kallikrein-associated fXIIa–fXIa and fXa-TF-VIIa components are inhibited. As a result, the primary enzymatic activities and complement cascades of the host defense mechanisms are largely disrupted (Chlastáková et al., 2021).

### Tick Salivary Molecules Inhibiting Host Coagulation Pathways

Tick salivary PIs are known to perform a vital role in coagulation, hemostatic inhibition, and platelet plugging (Šimo et al., 2017; Martins et al., 2020). Tick anticoagulant proteins (TAP; Kunitz-type family), including Om44 and TAI proteins derived from *Ornithodoros moubata*, were the first protease inhibitors discovered to specifically inhibit fXa (Waxman et al., 1990; Waxman and Connolly, 1993). TAI also blocks platelets’ adhesion to collagen (Karczewski et al., 1995), and Om44 blocks the P-selectin/PSGL-1 interaction, presumably preventing the leucocytes and platelets from adhering to vessel walls, which allows ticks to finish their blood sucking (Díaz-Martín et al., 2015). A TAP-like protein was also recognized in *O. savignyi* sialome that inhibited fXa and bound both exocites and active site of thrombin (Joubert et al., 1998). *In vitro* analysis of the recombinant tick anticoagulant peptide derived from *O. moubata* reduces TF/fVIIa-dependent thrombus formation (Ørvim et al., 1995). Ornithodorin of *O. moubata* is a highly effective and particularly selective thrombin inhibitor that specifically binds thrombin’s active site (N terminus) and exosite I (C-terminal helix) (Van de Locht et al., 1996). In addition, TAP derived from *O. moubata* interfere its tripeptide-containing amino terminal regions (Tyr-Asn-Arg) with both exocites and active site of thrombin. However, AsKunitz inhibition of thrombin’s activity might be due to the interaction of 2 cysteine components in the carboxy terminal region with exosite I only (Costa et al., 2021). The *Amblyomma variegatum* saliva protein, variegin, displays its C-terminal tail and directly binds thrombin’s active site to exosite I (Koh et al., 2007). *A. variegatum*-derived avathrin (homologous to variegin) is comprised of 32 residues and a competitive inhibitor that interacts with the thrombin active site and exosite I (Iyer and Goodman, 2019). *Rhipicephalus* (*Boophilus*) *microplus*-derived BmAP, microphilin, and boophilin (Horn et al., 2000; Ciprandi et al., 2006) can inhibit the host thrombin by binding the active site to exosite I. Additionally, boophilin also inhibits the activities of plasmin and trypsin (Macedo-Ribeiro et al., 2008). A fraction AV 16/3 of the salivary gland extracted from *A. variegatum* showed platelet aggregation, blood coagulation, and antithrombin activity in human platelets (Kazimirova et al., 2002). Salivary madanin 1 and 2 derived from *Haemaphysalis longicornis* bind anion-binding exosite 1 of the host thrombin, which inhibits the transformation of fibrinogen to fibrin by thrombin, fV and fVIII activation, and platelet aggregation without affecting its amidolytic activity (Iwanaga et al., 2003). Haemathrin 1 and 2 in the salivary gland of *Haemaphysalis bispinosa* specifically inhibit thrombin by cleaving its C-terminal end (Brahma et al., 2017). *A. sculptum*-derived amblyomin-X and *Ixodes scapularis-*derived Ixolaris, ixonnexin, and penthalaris salivary molecules have been proven to be inhibitors of fXa activation by inhibiting tissue factor fVIIa-Xa cascade coagulation (Francischetti et al., 2002; Francischetti et al., 2004; Chudzinski-Tavassi et al., 2010; Carneiro-Lobo et al., 2012; De Oliveira et al., 2012). The calcium-binding protein, longistatin, derived from *Hae. longicornis* that acts as an anticoagulant and plasminogen activator, hydrolyzes fibrinogen and delays the formation of fibrin clots (Anisuzzaman et al., 2010; Anisuzzaman et al., 2011). It also binds receptors for advanced glycation end products, mediating the stimulation of host immune cells, as a result, tempering the inflammatory and immunological responses of the host initiated due to tick bite (Anisuzzaman et al., 2014). The *Hae. longicornis* also secretes chimadanin in saliva that binds the active site of thrombin, inhibiting blood coagulation (Nakajima et al., 2005). Haemaphysalin, which binds to fXIIa and kallikrein BmTI-A, performs a crucial role in the inhibition of blood coagulation (Kato et al., 2005). *A. sculptum* secretes As8.9kDa, AsBasicTail, and AsKunitz; the former two prevent host trypsin and thrombin while inhibiting enzymatic activity derived from fXa, whereas AsKunitz inhibits only thrombin (Costa et al., 2021; Van Oosterwijk and Wikel, 2021). IxscS-1E1I of *I. scapularis* specifically inhibits thrombin, trypsin, cathepsin G, and fXa. It also inhibits adenosine diphosphate, thrombin’s induced platelet aggregation, and delayed the duration of plasma clotting (Ibelli et al., 2014). Other *I. scapularis* salivary proteins—such as penthalaris, metalloprotease, IsSMase, prostacyclin ISL1373, and ISL929—also modify blood coagulation (Ribeiro and Mather, 1998; Dai et al., 2010; Ibelli et al., 2014; Wang et al., 2016; Regmi et al., 2020). It is clearly demonstrated that the inhibitory activities and downstream dynamic responses of potent coagulant mediators against salivary molecules have an effective role in a tick’s success in blood-feeding. Approaches must be directed to design a rationale drug using an anticoagulant mediator for diverse clinical applications.

Salivary metalloproteases of *I. ricinus* and *I. scapularis* promote the delay of wound healing and angiogenesis through binding the cascade fibrinogen and fibrin and are involved in tissue disruption by digesting structural components (Francischetti et al., 2003; Decrem et al., 2008; Ali et al., 2014; Ali et al., 2015a; Ali et al., 2015b). In *I. ricinus*, metalloproteases interact with fXIIa, fXIa, and kallikrein mediators (Decrem et al., 2008). HLTnl derived from *Hae. longicornis* is a competitive inhibitor of the vascular endothelial growth factor receptor, thus delaying healing and angiogenesis (Fukumoto et al., 2006). Ixonnexin-mediated plasmin generation assists the interaction of lysine-binding sites of kringle domain(s) of plasminogen with t-PA, which promotes fibrinolysis. This protein also inhibits FeCl3-induced thrombosis in mice (Assumpção et al., 2018). Ixonnexin and salp14 derived from *I. scapularis* specifically bind plasmatic zymogen factor X, which impairs its binding with plasmatic or immobilized heparin, resulting in the inhibition of prothrombin-to-thrombin conversion (Narasimhan et al., 2002; Monteiro et al., 2005; Batista et al., 2010; Branco et al., 2016; Assumpção et al., 2018). Ixolaris inhibits the fVIIa/TF complex and impairs fXa binding to Sepharose-immobilized heparin by binding thrombin’s fXa heparin-binding exosite (Monteiro et al., 2005). Amblyomin-X suppress the formation of tumor growth and new blood vessels (angiogenesis) (Carneiro-Lobo et al., 2009); it also inhibits prothrombinase and tenase activities by hydrolyzing host trypsin and plasmin (substrate for trypsin and plasmin) (Branco et al., 2016). AamS6, AamAV422, and serpin19 derived from *A. americanum* interact with fXa and XIa trypsin, plasmin, T cells, and DCs and inhibit thrombin-initiated fibrin formation (Šimo et al., 2017) **(** **Table 1** **)**. HT-1, HT-3, and HT-12 in the salivary secretion of *I. holocyclus* target presynapses by inducing muscle paralysis that inhibits the dependence of transmitter release on extracellular calcium (Chand et al., 2016). Multiple tick salivary proteins have been identified and tested in the last few decades, but the molecular mechanism behind their host immunomodulation features is largely unknown (Bensaoud et al., 2019a). The impact of these molecules on tick–host interaction requires in-depth studies at the molecular and cellular levels to validate and develop candidates for anti-tick vaccine development.

**Table 1:** Tick-derived immunomodulatory molecules and their effects and interaction with host immune responses.

Tick salivary molecules with immunomodulatory functions	Effect	Interaction with host cellular immune response	Host used as experimental model	Tick species	References
AAS27	Anti-inflammation	Trypsin and serine protease inhibitor (trypsin-like)	Rabbit	*A. americanum* and *I. scapularis*	Tirloni et al., 2019
AamS6	Blood anticoagulation, platelet aggregation, and slowing of plasma clotting	Plasmin, papain, elastase, chymase	Rabbit	*A. americanum*	Mulenga et al., 2013a
AamAV422	Blood anticoagulation, antihemostasis, and anti-complementation	fXa and fXIa, plasmin, trypsin, T cell, DCs, and inhibition of thrombin-initiated fibrin formation	Rabbit	*A. americanum*	Mulenga et al., 2013b
Calreticulin	Blood anticoagulation	Host immunosuppression or antihemostasis	**-**	*A. americanum*	Jaworski et al., 1995
Serpin 19	Blood anticoagulation, antihemostasis	Factors Xa and XIa, trypsin, plasmin, T cell, and DCs	Rabbit	*A. americanum*	Kim et al., 2015a
AamIGFBP-rP1, AamIGFBP-rP6S, and AamIGFBP-rP6L	Innate host defenses	Provocation of an antibody response	Rabbit	*A. americanum, I. scapularis, R. microplus, R. appendiculatus*, and *A. variegatum*	Mulenga and Khumthong, 2010; Radulović et al., 2015
AAS19	Antibody titers provoked, antihemostasis, anticoagulant proteins produced, slowing of clotting in recalcification and thrombin time assays	Chymotrypsin, fIXa, fXa, fXIa, fXIIa, plasmin, trypsin, thrombin, tryptase	Rabbit	*A. americanum*	Kim et al., 2015a Kim et al., 2016
AAS 41 and 46	Inflammation defense	Chymase and chymotrypsin	Rabbit	*A. americanum*	Kim et al., 2020
Amregulin	Innate immune responses, anti-inflammation	TNF-α, IL-1, IL-8, IFN-γ	Mouse	*A. variegatum*	Tian et al., 2016
Variegin	Anticoagulant activities	C-terminus binding of exosite	Rabbit	*A. variegatum*	Koh et al., 2007
Avathrin	–	Thrombin active site and exosite I	–	–	Iyer and Goodman, 2019
SGE, Fraction AV 16/3	Platelet aggregation; Blood anticoagulation	Antithrombin effect on human blood platelets with hirudin-like activity	Rabbit	*A. variegatum*	Kazimirova et al., 2002
Amblyomin-X	Blood anticoagulation	Inhibition of fXa, prothrombinase, and tenase activities; substrate for plasmin and trypsin	Mouse	*A. cajennense* and *A. sculptum*	Branco et al., 2016
Sculptin	–	Inhibition of thrombin activity	–	*A. sculptum*	Iqbal et al., 2017
Monogrin	Platelet aggregation	Binding of platelets’ fibrin receptors with the RGD motif	–	*Argas monolakensis*	Mans et al., 2008
Prostaglandin E2 (PGE2)	Wound healing/innate immunity, angiogenesis	Regulation of MPs and fibroblast migration	Rabbit	*Dermacentor variabilis*	Poole et al., 2013
Da-P36	Acquired immune responses, immunosuppression	IgG	Rabbit	*D. andersoni*	Bergman et al., 2000
Heme lipoprotein	Inflammation	Binds galactose and mannose	Mouse	*D. marginatus*	Dupejova et al., 2011
Variabilin	Platelet aggregation	Antagonism of the fibrinogen receptor glycoprotein IIb-IIIa (GPIIb-IIIa, aIIbβ3); binding of platelets’ fibrin receptors with the RGD motif	Rabbit	*D. variabilis*	Wang et al., 1996
DsCystatin	Immunosuppression	Cathepsins L and B, decreased expression of CD80 and CD86	Mouse	*D. silvarum*	Sun et al., 2018
*Hae. longicornis* serpin-1 (HLS1)	Tick feeding	T cells and DC	Rabbit	*Hae. longicornis*	Sugino et al., 2003
HL-p36	Acquired immune responses, immunosuppression	Inhibition of IL-2, IL-12, and TNF-α expression		*Hae. longicornis*	Konnai et al., 2009
HlSerpin-a and HlSerpin-b	Suppression of inflammatory cytokines	NF-a, IL-6, and IL-1b bone-marrow-derived MPs (BMDMs) or mouse bone-marrow-derived dendritic cells (BMDCs)	Mouse	*Hae. longicornis*	Wang et al., 2020
Haemangin	Wound healing/angiogenesis and persistent blood-feeding	Vascular endothelial cell proliferation and induction of apoptosis	Rabbit	*Hae. longicornis*	Islam et al., 2009
Longicomin; Madanin 1 and 2	Platelet aggregation; Blood anticoagulation	Increased intracellular ca^2+^; Binding to exosites	Rabbit	*Hae. longicornis*	Cheng et al., 1999
HLTnI	Angiogenesis	Inhibition of growth factor (vascular endothelial)	–	*Hae. longicornis*	Fukumoto et al., 2006
HLS2	Coagulation, immunization of rabbits, increased tick death rate	Inhibition of thrombin activity	Rabbit	*Hae. longicornis*	Imamura et al., 2005
HL 34	Blood-feeding and oviposition	–	Rabbit	*Hae. longicornis*	Tsuda et al., 2001
Chimadanin	Blood anticoagulation	Inhibition of thrombin’s active site	Rabbit	*Hae. longicornis*	Nakajima et al., 2006
Haemaphysalin	Blood anticoagulation	Binding to fXIIa and kallikrein; BmTI-A	–	*Hae. longicornis*	Kato et al., 2005
Longistatin	Blood anticoagulation and feeding	Activation of plasminogen	Rabbit	*Hae. longicornis*	Anisuzzaman et al., 2011
Sialostatin tetraspanin CD63	**-**	CD4+ T-lymphocyte	Rabbit	*Hae. bispinosa* and *H. anatolicum anatolicum*	Boppana et al., 2004
Salivary gland extracellular vesicles (EVs)	Skin immunity and feeding	Murine bone-marrow-derived MPs, F4/80^+^ murine MPs, CD11b^+^ human MPs, dendritic epidermal T cells, upregulated γδ T cells	Mouse	*Hae. longicornis; I. scapularis*	Nawaz et al., 2020; Chávez et al., 2021
Haemathrin 1 and 2	–	Prevention of thrombins	–	*Hae. bispinosa*	Brahma et al., 2017
Enolase	Blood anticoagulation, anti-inflammation, and antihemostatic activities	Plasminogen receptor, stimulation of fibrinolysis	–	*Hae. flava*	Xu et al., 2016
Migration inhibitory factor homolog	Innate immune responses, inflammation, tumor growth, and angiogenesis	Inhibition of monocyte migration	Rabbit	*Hae. longicornis*	Jaworski et al., 2001
BIF	Acquired immune responses	Inhibition of B lymphocyte proliferation	Mouse	*H. asciaticum*; *I. ricinus*	Hannier et al., 2004; Yu et al., 2006
Hyalomin A and B	Innate immune responses, immunoregulation	TNF-α, interferon, and monocyte chemotactic protein-1	Mouse	*H. asciaticum*	Wu et al., 2010
HA24		Binding to histamine		*H. asiaticum*	Wang et al., 2016
Hyalomin-1	Blood anticoagulation, antiplatelet aggregation	Thrombin inhibition, anticoagulation activity	Mouse	*H. marginatum rufipes*	Jablonka et al., 2015
P5	–	Thrombin inhibition	–	*H. dromedarii*	Iqbal et al., 2017
Ixolaris	Blood anticoagulation, tissue-factor-pathway inhibitor (TFPI) homologue, extrinsic pathway	Inhibition of fVIIa/TF complex	Mouse	*Ixodes scapularis*	Francischetti et al., 2002; De Oliveira et al., 2012
Ixonnexin	Blood anticoagulation	Inhibition of the fXa	–	*I. scapularis*	Assumpção et al., 2018
Salp25D	Salivary protein, antioxidant (acquired immune responses)	Scavenging of ROS at the vector–pathogen–host interface	Mouse	*I. scapularis*	Narasimhan et al., 2007
tHRF B	Induction of histamine secretion	Basophils	Mouse	*I. scapularis*	Dai et al., 2010
TSLPI	Lectin complement cascade	Impairment of chemotaxis and neutrophil phagocytosis	Mouse	*I. scapularis*	Schuijt et al., 2011
Penthalaris	Anticoagulation, TFPI homologue	Inhibition of fVIIa/TF complex	Rabbit	*I. scapularis*	Francischetti et al., 2004
Salp14	Anticoagulation	Inhibition of the active site	Rabbit	*I. scapularis*	Narasimhan et al., 2002
TIX-5 (formerly known as P23)	Anticoagulation	Inhibition of fV activation through fXa	Rabbit	*I. scapularis*	Schuijt et al., 2013
Metalloprotease	Anticoagulation, feeding system	Degradation of fibrinogen and fibrin	Rabbit	*I. scapularis*	Francischetti et al., 2003
ISL929	Anti-inflammation and antihemostasis	Effecting neutrophils to downregulate production of superoxide and β2-integrins	Mouse	*I. scapularis*	Guo et al., 2009
tHRF	Blood-feeding and pathogen transmission assistance	Basophils, MCs, histamine/release	Rabbit	*I. scapularis*	Dai et al., 2010
ISAC	Complement system	Dissociation of C3 convertase	Rabbit	*I. scapularis*	Valenzuela et al., 2000
Salp20	Complement system	Binding of properdin and dissociation of C3bBbP, the active C3 convertase	Rabbit and mouse	*I. scapularis*	Tyson et al., 2007; Hourcade et al., 2016
Salp15	Immunosuppressive, facilitation of pathogen transmission, secretion of salivary protein (acquired immune responses), complement pathway	Inhibition of CD4+ T-cell activation; decrease of IL-6, TNF-α, and IL-12p35; CD4 binding; and IL-2 inhibition of neutrophil influx; dendritic cells	Rabbit and mouse	*I. scapularis*	Anguita et al., 2002; Ramamoorthi et al., 2005; Garg et al., 2006; Dai et al., 2009
IxscS-1E1	Blood anticoagulation, platelet aggregation	Thrombin, trypsin, cathepsin G, fXa to inhibited platelet aggregation and plasma clotting	Rabbit	*I. scapularis*	Ibelli et al., 2014
Sialostatin L and L2	Anti-inflammation, immunomodulation	Decrease of CD80/86, IL-12p70, TNF- α, and MHC II Ii processing; interaction of cytotoxic T lymphocyte-impaired IFN- and IL-17 with cytokines and dermis; annexin A2	Rabbit	*I. scapularis*	Kotsyfakis et al., 2006; Wang et al., 2016
Tryptogalinin	Immunomodulation	Inhibition of α-chymotrypsin, β-tryptase, β-trypsin, elastase, matriptase and plasmin	–	*I. scapularis*	Valdés et al., 2013
IsSMase	Adaptive immunity	Increased IL-4 and CD4+ T-cells	Mouse	*I. scapularis*	Alarcon-Chaidez et al., 2009
Prostacyclin ISL 1373	Vasodilation/vasoconstriction, tick feeding, hemostasis, angiogenesis	Binding to basophils, inducing the release of histamine; inhibition of microvascular endothelial cell (MVEC) proliferation	Rabbit	*I. scapularis*	Ribeiro and Mather, 1998; Francischetti et al., 2005a; Guo et al., 2009; Dai et al., 2010
PGE 2	Immune and inflammatory responses	Inhibition of IL-12 and TNF-α; CD40 differentiation	Mouse	*I. scapularis*	Sa-Nunes et al., 2007
Ipis-1	Inhibited proliferation and IFN-γ production of bovine PBMPs	Modulation of CD14+ cell activation	Rabbit	*I. persulcatus*	Toyomane et al., 2016
Salp15 Iper-1, Salp15, Iper-2	Acquired immune responses	(CD)4+ T-cells through the repression of T-cell receptor (TCR)-triggered calcium fluxes and interleukin (IL)-2 production	Mouse	*I. persulcatus*	Anguita et al., 2002; Mori et al., 2010
IP defensin 1 (IPDef1) and IR defensin 2 (IRDef2)	Pruritogenesis	Induction of MCs to produce cytokines	Mouse	*I. persulcatus*	Li et al., 2021
Salp16 Iper1, Salp16 Iper2	Innate immune responses, blood-feeding	Neutrophils, IL-8	Mouse	*I. persulcatus*	Hidano et al., 2014; Liu et al., 2014
IL-2-binding protein	Acquired immune responses	T-cell inhibition	Mouse	*I. scapularis*	Gillespie et al., 2001
PGE 2	Immunomodulation	Inhibition of IL-12 and TNF-α CD40 differentiation	Mouse	*R. sanguineus*	Oliveira et al., 2011
Evasin-1, 3, and 4	Innate immune responses, anti-inflammation	CXCL1, CXCL8, CCL3, CCL18, CCL4, CCL11, and CCL5	Mouse	*R. sanguineus*	Frauenschuh et al., 2007; Déruaz et al., 2008
Ado and PGE2	Innate immune responses	Inhibition of IL-12p40 and TNF-α; decrease activation of CD40 Raf-1/MEK	Mouse	*R. sanguineus*	Hovius et al., 2008a; Oliveira et al., 2011
Calcaratin	Blood anticoagulation	Chromogenic substrates S-2238 for thrombin and S-2765	–	*Boophilus calcaratus*	Motoyashiki et al., 2003
Ixodegrin	Platelet aggregation	Binding of platelets’ fibrin receptors with the RGD motif	**-**	*I. scapularis* and *I. pacificus*	Francischetti et al., 2005b
TSLP1	Inhibition of the host lectin complement pathway and neutrophil chemotaxis	Mannose-binding lectin complement pathway	Rabbit	*I. scapularis* and *I. ricinus*	Wagemakers et al., 2016
Ir-CPI	Anticoagulation	Inhibition of intrinsic coagulation pathways fXIIa, fXIa, and kallikrein	Mouse	*I. ricinus*	Decrem et al., 2009
*Ixodes ricinus* serine protease inhibitor (IrSPI)	Immunomodulatory action	Blocking of CD4+ T lymphocyte proliferation and pro-inflammatory cytokine secretion from splenocytes and MPs	Rabbit and mouse	*I. ricinus*	Blisnick et al., 2019
Iris and Iris2	Anti-inflammation, blood coagulation, and fibrinolysis	Thrombin, elastase, t-PA, fXa, trypsin; suppression of T-cell and splenocyte proliferation; alteration of cytokine secretion by PBMC; binding of monocytes/MPs and inhibition of TNF secretion; MPs and IFN-γ	Rabbit and mouse	*I. ricinus*	Kim et al., 2015b
Iripin-3	Modulation of the adaptive immune response	Spleen cells, weakened proliferation of CD4+ T lymphocytes, suppression of the T helper type 1 immune response, induction of regulatory T-cell differentiation, reduced creation of pro-inflammatory cytokine interleukin-6 by bone-marrow-derived MPs stimulated by lipopolysaccharide	Mouse	*I. ricinus*	Chlastáková et al., 2021
miRNA	Inflammation, host homeostasis, and pain sensing	–	Rabbit	*I. ricinus*	Hackenberg et al., 2017
IRS-2	Inflammation inhibition, induced platelet aggregation, inhibited Th17 differentiation*/*blood coagulation/innate immune responses	T-cell differentiation, T17 cell, chymase and cathepsin G, MCs, protease-4, thrombin, trypsin, a-chymotrypsin reduction in production of IL-6 in DCs slow‐binding classical inhibitor, Inhibition of neutrophil migration	Mouse	*I. ricinus*	Chmelař et al., 2011
RNAi-silenced *N*-ethylmaleimide-sensitive factor attachment receptor (SNARE) genes (*synaptobrevin2* and *vamp33*)	Diminished feeding, skin immunity	Adaptive T-helper-2 (Th2), γδ T cells, DCs epidermal T cells	Mouse	*I. scapularis*	Chávez et al., 2021.
Ir-LBP	Innate immune responses	Inhibition of neutrophil chemotaxis	Mouse	*I. ricinus*	Beaufays et al., 2008
B cell–inhibitory proteins (BIPs)	Acquired immune responses	Prevention of B-cell activation	Mouse	*I. ricinus*	Hannier et al., 2004
Iristatin	Immunomodulation	Inhibition of the activity of cathepsins C and L and reduction in the production of IL-9, IL-2, IL-4, and IFN-γ MCs and MPs	Rabbit	*I. ricinus*	Kotál et al., 2019
Metalloproteases	Wound healing/angiogenesis	Binding to fXIIa, fXIa, and kallikrein	Mouse	*I. ricinus*	Decrem et al., 2008
Salp15-like protein	Acquired immune responses	Inhibition of IL-10 production		*I. ricinus*	Liu et al., 2014
Irac I and II and Isac; Paralogues	Complement alternative pathway, blood-feeding	Dissociation of C3 convertase	Rabbit	*I. ricinus*	Schroeder et al., 2007
64TPR	Inflammation, immune responses, and feeding	CD4^+^ T cells	Mouse	*I. ricinus*	Labuda et al., 2006
Apyrase	Platelet aggregation	Hydrolyzation of ATP and ADP to inactive AMP	**-**	*Ornithodoros* spp.; *I. scapularis*	Ribeiro et al., 1985; Mans et al., 1998
Om44	Protective anti-tick immune responses	Inhibition of fXa	Pig	*O. moubata*	García-Varas et al., 2010
Moubatin	Platelet aggregation	Lipocalin, binding of Thromboxane A2 (TXA2)	–	*O. moubata*	Waxman and Connolly, 1993; Mans and Ribeiro, 2008
TAI	Platelet aggregation	Inhibition of fXa; inhibition of platelets and adhesion to collagen	–	*O. moubata*	Karczewski et al., 1995
Disagregin	Platelet aggregation	Thrombin, the thrombin receptor peptide, agonists, epinephrine, collagen, platelet-activating factor	–	*O. moubata*	Van de Locht et al., 1996
Ornithodorin	Blood anticoagulation	Binding to both exosites and active sites; inhibition of thrombin	–	*O. moubata*	Waxman et al., 1990
Enolase	Blood anticoagulation	Reception of plasminogen; stimulation of fibrinolysis	Rabbit	*O. moubata*	Diaz-Martin et al., 2013
TAP	Blood anticoagulation	Binding to both exosites and active sites	–	*O. moubata*	Karczewski et al., 1994
OmCI	Complementation	C5a disruption by human classical and alternative C5 convertases	–	*O. moubata*	Nunn et al., 2005
OP-15 and OP-16	Anti-complementation activity, platelet aggregation inhibition	–		*O. parkeri*	Francischetti et al., 2008
Savignygrin	Platelet aggregation	Binding of platelets’ fibrin receptors with the RGD motif, Kunitz	–	*O. savignyi*	Mans et al., 2002
Savignin	Platelet aggregation	Binding exosites and active sites of thrombin	–	*O. savignyi*	Nienaber et al., 1999
TAP-like protein	Platelet aggregation	Binding exosites and active sites, inhibition of fXa	–	*O. savignyi*	Joubert et al., 1998
TSGP2 and TSGP3	Neutrophil, platelet aggregation, complementation, vasoconstriction	Scavenging of TXA2 and leukotriene B4; C5 complement targeting activity	–	*O. savignyi*	Mans and Ribeiro, 2008
	Complementation	Interaction with complement C5		*O. kalahariensis*	
64TRP	Protection provided against TBEV infection, cement protein involved in attachment and feeding	CD4+, CD8+ T-cell infiltration and expression of ICAM-1, Ia antigens, IL-1 alpha, and TNF-α	Mouse, hamster, rabbit, and guinea pig	*R. appendiculatus*, and *I. ricinus*	Labuda et al., 2006; Havlíková et al., 2009
RAS-1 and 2; RAS-3 and 4	Enhanced tick mortality rate	–	–	*R. appendiculatus*	Imamura et al., 2006; Imamura et al., 2008
Histamine-binding proteins (HBPs) RaHBP (M) and RaHBP(F)	Innate immune responses; Anti-inflammation	Lipocalins, histamine‐binding, anti-inflammatory, basophils, MCs/binding of histamine	Cow and mouse	*R. appendiculatus* and *D. reticulatus*	Sangamnatdej et al., 2002; Štibrániová et al., 2019
TdPI	Innate immune responses	MCs; inhibition of tryptase	Mouse	*R. appendiculatus*	Paesen et al., 2007
65 kDa protein	Blood anticoagulation	Inhibition of thrombin-initiated fibrin formation	Rabbit	*R. appendiculatus*	Limo et al., 1991
Japanin	Acquired immune responses, adaptive immunity	Dendritic cells inhibition of DCs differentiation	–	*R. appendiculatus*	Preston et al., 2013
RaCI	Complement inhibition	Activation of C5 convertase	–	*R. appendiculatus*	Silva et al., 2016; Jore et al., 2016
RHS-1 and RHS-2	Antiplatelet activity	Chymotrypsin, thrombin, fXa to anticoagulation activity, RNAi-disrupted tick feeding	–	*R. haemaphysaloides*	Yu et al., 2013
Rhipilin-1 and Rhipilin-2	Inhibition of platelet aggregation and blood coagulation	TFPI inhibition of serine protease trypsin and elastase; anticoagulation activity	Rabbit	*R. haemaphysaloides*	Gao et al., 2011; Cao et al., 2013
RH36	Immunosuppression	Suppression of T lymphocyte proliferation; inhibition of the expression of cytokines such as IL-2, IL-12, and TNF-α	–	*R. haemaphysaloides*	Wang et al., 2017
RmS-3	Reduced platelet aggregation, impaired antibody reproduction	Chymotrypsin, cathepsin G, elastase, chymase	–	*R. microplus*	Tirloni et al., 2014; Tirloni et al., 2016
RmS-1 and RmS-6	Reduced platelet aggregation, impaired antibody reproduction	Trypsin, plasmin, fXa, fXIa, chymotrypsin	–	*R. microplus*	Tirloni et al., 2014; Tirloni et al., 2016
RmS-15	Antihemostasis	Thrombin to delayed plasma clotting	Cow	*R. microplus*	Tirloni et al., 2014
RmS-17	Reduced platelet aggregation	Plasmin, trypsin, chymotrypsin, cathepsin G, fXIa to delayed plasma clotting	–	*R. microplus*	Tirloni et al., 2014; Tirloni et al., 2016
rSerpin	Enhanced tick blood-feeding duration and mortality and reduced tick engorgement and egg mass	T cells and DC	–	*R. microplus*	Jittapalapong et al., 2010
BmAP	Blood anticoagulation	Inhibition of active sites and exosites	–	*R. microplus*	Horn et al., 2000
Microphilin	Blood anticoagulation	Thrombin inhibition, exosite binding only, blocking of thrombin at exosite I, inhibition of coagulation and thrombin-induced platelet aggregation	Rabbit	*R. microplus*	Ciprandi et al., 2006

Hyalomin 1 from *Hyalomma marginatum rufipes* and Hyalomin A and B from *H. asciaticum* deactivate host factor XI (fXI) and prevent thrombin activity by binding its active site and exosite I (Jablonka et al., 2015). *Hae. longicornis* longistatin binds to the V domain of receptor for advanced glycation end products, thereby inhibiting tissue inflammation (Anisuzzaman et al., 2010; Anisuzzaman et al., 2014). An anticoagulant TIX-5 (formerly known as P23) in the secretome of *I. scapularis* specifically prevents fV activation mediated by fXa that activates fV by fXa by involving the B-domain of fV (Schuijt et al., 2011; Aleman and Wolberg, 2013; Schuijt et al., 2013). The Ir-CPI, with one Kunitz domain interference with fXIIa, fXIa, and kallikrein, inhibits the intrinsic-specific coagulation pathway (Decrem et al., 2009). *R. calcaratus*, calcaratin, assists in the anticoagulation of blood by inhibiting thrombin S-2238 and fXa S-2765 chromogenic substrates (Motoyashiki et al., 2003). Lipocalin proteins are most commonly produced in tick SGs and have been implicated in host inflammation modulation by scavenging histamine and serotonin (Sangamnatdej et al., 2002; Mans et al., 2008), targeting platelet aggregation and the complement system, as well as being involved in toxicoses and as allergens (Hilger et al., 2005; Nunn et al., 2005). *A. sculptum* sculptin (Iqbal et al., 2017) and *H. dromedarii* P5 (Ibrahim and Masoud, 2018) have shown inhibition of thrombin activity.

### Antiplatelet Aggregation (Tick Salivary Molecules Inhibiting Host Platelet Aggregation)

Tick-derived inhibitors target the enzymes that stimulate platelet aggregation, such as cathepsins G (Chmelař et al., 2011), C, L, S, V, and H (Chmelař et al., 2017); chymase (Chmelař et al., 2011); elastase (Cao et al., 2013; Valdés et al., 2013); and tryptase (Cao et al., 2013; Mulenga et al., 2013a). Platelets show three β1 integrins—α2β1, α5β1, and α6β1—and two β3 integrins—αIIbβ3 and αVβ3 (Joo, 2012)—which are inhibited by tick salivary molecules or bind platelet activators like ADP, serotonin, or thromboxane, thus hindering the mechanism of platelet aggregation (Chmelař et al., 2012). *I. scapularis* sialostatin L and L2 have been identified as specifically inhibiting cathepsins S, C, L, and papain (Kotsyfakis et al., 2006). Sialostatin L is an effective inhibitor of lysosomal cysteine cathepsins X and V (Štibrániová et al., 2019), and sialostatin L2 specifically inhibits cathepsin B and H (Kotsyfakis et al., 2010). RHcyst-2 derived from the saliva of *R. haemaphysaloides* inhibits cathepsin S (Wang et al., 2015). Omc2 from *O. moubata* inhibits cathepsins S and L (Salát et al., 2010). Iristatin of *I. ricinus* and BrBmcys2b and BrBmcys2c of *R. microplus* suppress host cathepsins C and L; however, BrBmcys2b also inhibits cathepsin B (Parizi et al., 2015; Parizi et al., 2018; Kotál et al., 2019). Iristatin diminish IL-2, IL-4, and IL-9, and IFN-γ-stimulated Th1 cells; IL-6 and IL-9; the production of nitric oxide by MPs; and anti-inflammatory cytokines IL-2 and IL-9 by Th9 cells and IL-4 by Th2 cells (Kotál et al., 2019). Salivary serpin Iris2 of *I. ricinus* inhibits cathepsin G and chymase to prevent inflammation (Kim et al., 2015b). Tryptogalinin secreted in the sialome of *I. scapularis* plays a wide variety of roles in the host immunomodulation by inhibiting α-chymotrypsin, β-tryptase, β-trypsin, elastase, matriptase, and plasmin and exerting a potentially extensive effect against several host enzymes and mast cell proteins (Valdes et al., 2013). *Hae. longicornis* Hlcyst-2 and Hlcyst-3 and *I. persulcatus-*derived JpIpcys2a, b, and c inhibit Cathepsin L and papain (Zhou et al., 2006; Rangel et al., 2017) **(** **Table 1** **)**. Haemangin has been recognized as a Kunitz inhibitor from the saliva of *Hae. longicornis* that intensely prevents trypsin, chymotrypsin, and plasmin and thus supports the inhibition of plasmin-dependent fibrinolysis and angiogenic cascades (Štibrániová et al., 2019).

**Figure 1:**
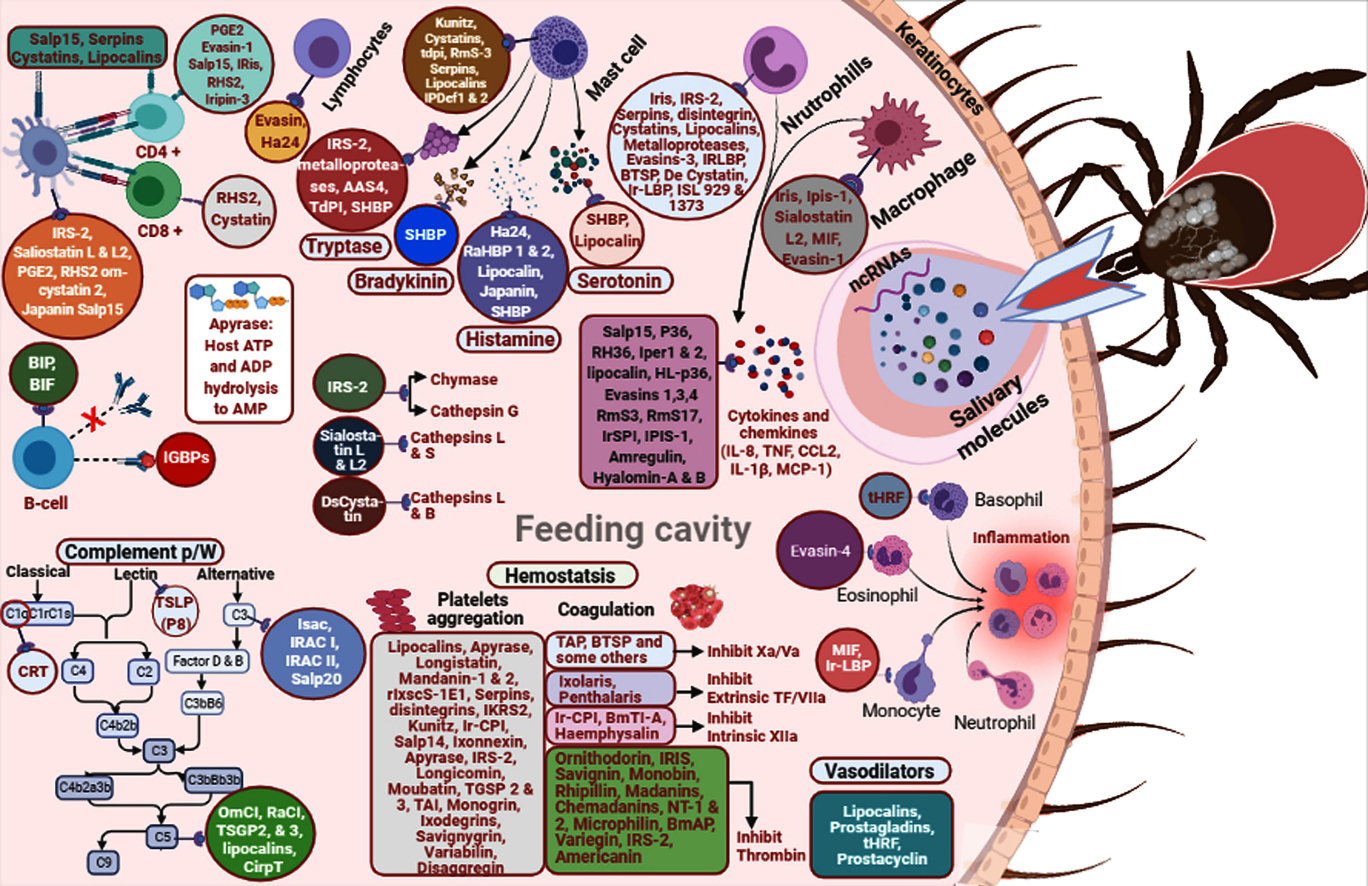
A diagram of tick salivary components involved in invasion of host responses.

The *R. appendiculatus*–derived protease inhibitor TdPI effectively inhibits human β-tryptase and trypsin, as well as human plasmin (Paesen et al., 2007). Enolase from *Haemaphysalis flava* and *O. moubata* has been found to affect blood coagulation and possess anti-inflammatory and antihemostatic activities (Xu et al., 2016). *R. microplus* serpins have shown specific inhibition of chymotrypsin (RmS-1, RmS-3, RmS-6), and thrombin (RmS-15) (Rodriguez-Valle et al., 2015). *I. scapularis* and *I. pacificus* ixodegrin bind fibrin receptors on platelets with the RGD motif that provokes platelet aggregation (Francischetti et al., 2005b). *In vivo* studies have shown that YY-39 from *I. pacificus* and *I. scapularis* plays a role in platelet and thrombosis modulations by decreasing adenosine diphosphates, platelet adhesion to soluble collagen, thrombin- and TXA2-induced platelet aggregation, and binding to purified GPIIb/IIIa (Tang et al., 2015). Tick saliva–derived anti-inflammatory molecules such as AAS27 and AAS41 from *A. americanum* and *I. scapularis* and IRS-2 from *I. ricinus* target chymase to inhibit the inflammatory response of the host (Tirloni et al., 2019; Kim et al., 2020). In addition, AAS27 inhibits inflammation by targeting plasmin and trypsin activities (Tirloni et al., 2019), and IRS-2 targets cathepsin G activity, thus involving the inhibition of host vasodilation (Chmelař et al., 2011). DsCystatin identified in *Dermacentor silvarum* salivary glands weakens the activities of host cathepsins L and B of pro-inflammatory cytokine IFNγ, TNFα, and IL6 expression, and TLR2 and TLR4 signaling pathways induced NFκB activation (Sun et al., 2018). *O. moubata*-derived salivary protein disagregin interacts with the host’s αIIbβ3 integrin receptor that blocks its binding site to the fibrinogen, thus diminishing collagen tissue factor–mediated platelet aggregation and thrombus formation (Karczewski et al., 1994; Van den Kerkhof et al., 2021). *D. variabilis* variabilin blocks the binding of GPIIb-IIIa, αIIbβ3, and vitronectin receptor αvβ3 (Wang et al., 1996). *O. savignyi* savignygrin binds the αIIbβ3 and dissociates fibrinogen from its receptor (Mans et al., 2002); FXal binds to both exosites and active sites of thrombin (Gaspar et al., 1996). Other savignins have been observed to be involved in complement activities, vasoconstriction, and platelet and neutrophil aggregation (Mans et al., 2002; Mans and Ribeiro, 2008). BmTI-A from *R. microplus* hinders angiogenesis *in vitro* in a vessel formation assay and blocks neutrophil elastase, trypsin, plasmin, and plasma kallikrein (Soares et al., 2016). *O. moubata* moubatin and TSGP3 inhibit collagen-induced platelet aggregation and bind to TXA2; they also mediate vasoconstriction in the rat aorta. TXA2 is inhibited by longicornin from *Hae. longicornis* (Cheng et al., 1999). TSGP2 and TSGP3 from *O. savignyi* saliva bind host leukotrienes B4 and TXA2 with high affinity (Mans and Ribeiro, 2008). *Argas monolakensis* monogrin binds with fibrin receptors on platelets with the RGD motif that prevents platelet aggregation (Mans et al., 2008). Iris (elastase inhibitor) of *I. ricinus* hampers the intrinsic pathway and platelet aggregation (Pham et al., 2021) and inhibits the activities of elastase, thrombin, t-PA, fXa, and trypsin (Prevot et al., 2006). AAS19 is an anticoagulant protein derived from *A. americanum* that inhibits the activity of thrombin, plasmin, trypsin, fXa, fIXa, fXIa, fXIIa, tryptase, and chymotrypsin (Kim et al., 2015a). *Dermacentor marginatus* heme lipoprotein is a carbohydrate-binding protein with mannose- and galactose-binding specificity inhibiting agglutination; therefore, the presence of heme lipoproteins might lower the quantity of free heme at the feeding site by inhibiting inflammation (Dupejova et al., 2011; Tirloni et al., 2014; Tirloni et al., 2015). AM-10 and AM-38 from *A. monolakensis* have shown high affinity for histamine and 5-HT binding at the feeding spot, thereby preventing inflammatory actions (Mans et al., 2008). High-affinity HBPs from the saliva of *R. appendiculatus* have also been explored, where these proteins independently bound H1, 2, and 3 membrane receptors of histamine secreted by host basophils (Paesen et al., 1999). TIL-domain inhibitors have been reported in the salivary secretion of *R. appendiculatus* and *I. ricinus* (De Castro et al., 2017). Growth factor binding proteins—AamIGFBP-rP6L, AamIGFBP-rP-1, and AamIGFBP-rP6S—have been identified from *A. americanum* (Bakshi et al., 2019; Bartíková et al., 2020). *R. haemaphysaloides* salivary proteins RHS-1 and RHS-2 have shown antichymotrypsin activity, while RHS-1 has shown anticoagulation activity, based on activated partial thromboplastin time (APTT). Both inhibitors have been found to be involved in the inhibition of thrombin and fXa (Yu et al., 2013). Rhipilin-1 and Rhipilin-2 isolated from *R. haemaphysaloides* inhibit platelet aggregation by inhibiting tissue factor pathway inhibitor (TFPI) and coagulant activities involving the inhibition of serine protease elastase and trypsin (Gao et al., 2011; Cao et al., 2013). Various tick salivary-secreted molecules involved in host invasion and interfering with host defense mechanisms are displayed in **Figure 1**.

### Tick Salivary Molecules Inhibiting the Complement Pathways of the Host

The complement cascade—including classical, lectin, and alternative pathways— is the first line of a host’s defense mechanisms that can be triggered by tick salivary molecules targeting the host’s enzymes, thereby disrupting complex assembly and downstream activation (Rosbjerg et al., 2017). The complement pathway plays a great role in controlling tissue injury and pathogen invasion when ticks have secreted several salivary effector proteins that specifically target C3 and C5 (Pham et al., 2021). The investigated anti-complementary features of tick salivary molecules include Isac, Salp9, and Salp20 from *I. scapularis*, and Irac I, Irac II, and IxACs from *I. ricinus* (Brossard and Wikel, 2004; Štibrániová et al., 2019). Among these, Isac, Irac-1 and -2, and Salp20 bind and displace properdin, thereby inhibiting C3 convertase formation (Hourcade et al., 2016; Silva et al., 2016); subsequently, dissociating C3 convertase complex (C3bBbP) from the alternative pathway prevents binding of the convertase to C3 (Francischetti et al., 2002; Monteiro et al., 2005). *O. moubata* OmCI and *R. appendiculatus* RaCI are C5 complement inhibitors that prevent its activation by C5 convertase, including MG1, MG2, and C5d domains and 5d, CUB, and C345c domains respectively (Nunn et al., 2005; Jore et al., 2016; Silva et al., 2016), probably inhibiting the rearrangement of the domains within C5, as it is necessary for activation to occur (Jore et al., 2016). Additionally, OmCI and its homologs bind complement C5 and leukotriene B4 (LTB4), inhibiting the induced inflammation (Roversi et al., 2013). C5 complement inhibitors have been reported from *R. appendiculatus*, *R. microplus*, *D. andersoni*, and *H. marginatum* (Silva et al., 2016); however, their anti-complement activity regarding binding to numerous sites at the C5 component remains to be investigated (Silva et al., 2016). *O. kalahariensis* TSGP2 and TSGP3 are comprised of a histidine residue His95 and a conserved βH-α2 loop in the sequences implicated in the interaction with complement C5. Both salivary components from *O. savignyi* have been reported to be responsible for the inactivation of the C5 mediator (Mans and Ribeiro, 2008). Saliva of *A. cajennense* is also involved in classical pathway inhibition; however, the inhibitor to date has not been identified (Franco et al., 2016; Silva et al., 2016). *I. dammini* saliva antagonizes anaphylatoxin and bradykinin, likely by the presence of a carboxypeptidase, and also blocks the deposition of C3b and the release of C3a (Andrade et al., 2005). A *R. microplus* salivary thiol-activated metalloendopeptidase has been shown to hydrolyse bradykinin and may have a role in relieving pain and other inflammatory signs at the tick bite site (Bastiani et al., 2002) OP-15 and OP-16 in *O. parkeri* from a sialome project have been identified (Francischetti et al., 2008). The molecular mechanisms of the classical pathway inhibition in *R. microplus* involve the binding of tick salivary calreticulin (C1-inhibitor (C1-INH)) to the C1 of the initially activated factor of C1qC1rC18 of the classical pathway, which in turn blocks the deposition of C4b (De Taeye et al., 2013); however, it does not activate the classical complement cascade in *A. americanum* (Kim et al., 2015b). The *I. scapularis* salivary lectin pathway inhibitor (TSLPI) prevents the binding of mannose-binding lectin and ficolins to their ligands, thus inactivating the cascade of the lectin pathway (Silva et al., 2016; Wagemakers et al., 2016; Vechtova et al., 2018; Coumou et al., 2019). AsKunitz and As8.9kDa are the first *A. sculptum* salivary inhibitory components of the classical pathway. Among these, As8.9kDa is known to inactivate the C3b factor, thus inhibiting the downstream cascade (Costa et al., 2021) **(** **Figure 1** **)**. Salivary proteins that inhibit binding of mannan-binding lectin to the polysaccharide mannan were characterized in *I. scapularis* (TSLPI, Salp1, and Salp9Pac) and *O. savignyi* (BSAP), resulting in the inactivation of the lectin complement pathway (Denisov and Dijkgraaf, 2021). Other complement pathway inhibitors and their molecular mechanisms require further studies to be properly elucidated.

### Host Cellular Response Modulating the Secretion of Tick Salivary Molecules

Cytokines and chemokines are the complement mediators that create a network and recruit leucocytes to site of the tick bite (Sangamnatdej et al., 2002). Cytokines are inhibited by salivary inhibitors (Cathepsin L and S proteins), and chemokines (CC and CXC) are bound by salivary evasins, thereby preventing the host’s inflammatory responses (Bhusal et al., 2020). Cathepsin L and S proteins diminish the phosphorylation of STAT-1 and STAT-2, thus effecting JAK/STAT signaling in DCs (Pham et al., 2021). PGE2 derived from *D. variabilis*, *R. sanguineus*, and *I. scapularis* inhibits DCs maturation and fibroblast migration and decreases CD40 Raf-1/MEK activation *in vitro* (Hovius et al., 2008a; Oliveira et al., 2011; Poole et al., 2013). Evasins from *R. sanguineus*—Evasin-1 (CCL18, CCL4, and CCL3), Evasin-3 (CCL1 and CCL8), and Evasin-4 (CCL11 and CCL5)—are responsible for the inhibition of chemokines (Frauenschuh et al., 2007; Guo et al., 2009). T-cell inhibitors have been found to either bind or inhibit their associated release or activation factors, including *I. ricinus*–derived IrSPI and Iris, *I. persulcatus* Ipis-1, *R. microplus* RmS-3 and RmS-17, and *I. scapularis* Salp15 (Ramamoorthi et al., 2005; Dai et al., 2009; Blisnick et al., 2019; Almazán et al., 2020). Iripin-3 from *I. ricinus* provides activity against the extrinsic blood coagulation pathway; moreover, this protein was shown to interfere with the adaptative immune response, since it reduces the production of interleukin-6 by MPs and the reduction of the T helper type 1 immune response (Chlastáková et al., 2021).

Similarly, tick salivary sialostatin L dampens antigen-mediating CD4+ proliferation and reduces the activation of interferon regulatory factor 4 signaling in MCs (Pham et al., 2021). Salp16 and Iper proteins are immunosuppressants that inhibit IL-8 activity, thereby impairing neutrophil chemotaxis (Sajiki et al., 2020). Salp14 inhibits T-cell proliferation and decreases the pro-inflammatory IL-6 and TNF-α production and secretion (Pham et al., 2021). Amregulin from *A. variegatum* inhibits the secretion of IL-1, TNF-α, IL-8, IFN-γ, and CXCL8 by LPS by stimulated rat splenocytes *in vitro* in a dose-dependent manner (Tian et al., 2016). Japanin immune-modulatory lipocalin derived from *R. appendiculatus* saliva was found to specifically reprogram DCs by blocking its differentiation from monocytes and altering the set of sequences, including pro-inflammatory, anti-inflammatory, transmembrane molecules, and cytokine secretion (Preston et al., 2013). HlSerpin-a and b derived from the saliva of *Hae. longicornis* suppress inflammatory cytokines by decreasing NF-a, IL6, and IL-1b bone marrow–derived MPs or mouse bone marrow–derived DCs (Wang et al., 2020).

*I. scapularis* and *D. andersoni* saliva histamine release factors were found to enhance histamine release and promote vasodilation upon binding to host basophils (Bhowmick and Han, 2020). Serotonin- and histamine-binding proteins (SHBPs) have been found in the saliva of *D. reticulatus*, simultaneously binding host serotonin and histamine (Sangamnatdej et al., 2002), and monotonin derived from *A. monolakensis* binds serotonin (Mans et al., 2008). *R. appendiculatus*-derived Ra-HBPs (male Ra-HBP1 and Ra-HBP3 and female Ra-HBP2) and Japanin have been identified as possessing a high affinity toward histamine binding. Functionally, Ra-HBP1 is a weak histamine binder as compared to Ra-HBP2 and 3, which display potent affinity to histamine (Štibrániová et al., 2019). *H. asiaticum-*derived HA24 binds specifically to histamine with a particular histamine-binding affinity demonstrated in a dose-dependent manner (Wang et al., 2016). Moubatin-like 3 stoichiometrically binds two histamine or one serotonin molecules (Mans and Ribeiro, 2008). MC migration inhibitory factor homologs have been isolated from the saliva of *A. americanum*, which plays a key role in the inhibition of migration of human MPs (Jaworski et al., 2001).

*I. scapularis* sialostatin L and L2 dampen antigen-mediated CD4+ proliferation, reduce the activation of interferon regulatory factor 4 signaling in MCs (Klein et al., 2015; Lieskovska et al., 2015), and influence the maturation of DCs by inhibiting IFN-β (Lieskovska et al., 2015). Three immunosuppressant salivary proteins—p36 from *D. andersoni* (Alarcon-Chaidez et al., 2003), HL-p36 from *Hae. longicornis* (Konnai et al., 2009), and RH36 from *R. haemaphysaloides*—blocked the T-lymphocyte proliferation *in vitro* assay (Wang et al., 2017). HL-p36 and RH36 directly inhibited the proliferation of many mitogen-stimulated cells *in vivo* and the expression of numerous cytokines such as IL-12, IL-2, and TNF-α (Kotsyfakis et al., 2006; Kotsyfakis et al., 2010). RHS2 can also inhibit CD4+ and CD8+ T-cell activation, leading to the inhibition of the host Th1 immune response.

### Host Humoral Immune Response to Tick Salivary Molecules

Host immune responses interacting with tick salivary molecules may impede the host-associated regulatory and\or signaling pathways, evolving different mechanisms such as the suppression of the host humoral response, which in turn enhances tick blood-feeding (Andrade et al., 2005). Host humoral immunity is inhibited by tick salivary components by disrupting the B cell–derived immune responses, such as their manufacturing of specific antibody inhibitors (Andrade et al., 2005). SGs of several ixodid tick species secrete a set of immunoglobulin-G binding proteins (IGBPs) (Andrade et al., 2005). B cell–inhibitor factors derived from *H. asciaticum asciaticum* provoke B-cell responses (Yu et al., 2006), and B cell–inhibitory proteins derived from *I. ricinus* inhibit B lymphocyte proliferation, while not affecting T lymphocytes (Hannier et al., 2004). Further investigation might be necessary to discover its probable effects on B-cell receptor signaling pathways and Toll-like receptors (TLRs) such as TLR1, TLR2, and TLR4. Limited reports on B cell inhibition and the suppression of B-cell antibody production are available. The discovery of novel drugs requires components that could be a functional template for targeting B cells and their associated immune pathways. For this purpose, B-cell inhibitors in tick sialome are an essential target for determining their biological activities in host immunomodulation.

### Tick Salivary-Derived Non-Proteinaceous Molecules Impairing the Host Immune System

Several tick salivary non-proteinaceous molecules have been identified, such as PGE2, prostacyclin, purine nucleoside adenosine, fatty acids, endocannabinoids, and recently discovered non-coding RNAs (ncRNAs), that induce host immunomodulation (Hermance et al., 2019). Among these, PGE2 was the first identified non-proteinaceous immunomodulatory component in tick sialome that recruits several inflammatory cells to the target site (Andrade et al., 2005). The PGE2 component was reported in saliva secreted by tick species of the following genera—*Rhipicephalus*, *Dermacentor*, *Ixodes*, and *Amblyomma*—potentially influencing the inhibition of T lymphocytes, B lymphocytes, and immune sentinel MPs (Hermance et al., 2019; Sajiki et al., 2020). Pro-inflammatory TNF-α, IL-12p40, and IL-10 by murine DCs are stimulated by saliva-derived non-proteinaceous purine nucleoside adenosine in *R. sanguineus* (Oliveira et al., 2011). Tick sialome-derived fatty acid organic amide, prostacyclin, and endocannabinoids have been reported to be involved in the enhancement of host vasodilation and analgesic and anti-inflammatory activities (Hackenberg et al., 2017). Tick-secreted saliva contains a set of enzymes that belong to the 5′-nucleotidase family, among whose enzymes tick salivary apyrases have been identified in the saliva of *O. savignyi*, *O. moubata*, *O. kalahariensis*, and *I. scapularis* (Ribeiro et al., 1985; Ribeiro et al., 1991; Mans et al., 1998; Valenzuela et al., 2002; Stutzer et al., 2009), which cleave to extracellular ATP and ADP to prevent platelet stimulation and aggregation. A molecular mechanism elucidated the linkage of the host’s immunomodulation with the rigorous activities of tick salivary prostaglandin E2 and purine nucleoside adenosine (Sá-Nunes et al., 2007; Oliveira et al., 2011).

Several transcribed mRNAs have been reported to show non-protein-coding parts but involving several cellular regulatory functions termed as ncRNAs (Jarroux et al., 2017). They are categorized into long non-coding RNAs and small non-coding RNAs having nucleotide ranges of <200 nucleotides and >200, respectively. They have been found to functionally participate in subverting the host’s defensive responses in the parasite–vector–host interface (Bensaoud et al., 2019b; Ahmad et al., 2021). Further investigation of long non-coding RNAs and small non-coding RNAs localization, purpose for secretion, and molecular mechanisms is necessary, as they are suggested to have a putative role in the regulation of gene expression and to disrupt the signaling between the host defense pathways in the host’s cells (Hackenberg and Kotsyfakis, 2018; Chávez et al., 2019). A wide range of extracellular vesicles (EVs) have been identified that contain molecules of modulating the host’s physiology and enabling the blood-feeding mechanism (Chávez et al., 2021). Previously, tick exosomes were suggested in the secretion of ncRNAs (Hackenberg and Kotsyfakis, 2018; Chávez et al., 2019), which was recently confirmed through the successful isolation from saliva and SGs of partially fed or unfed ixodid ticks (Zhou et al., 2020; Chávez et al., 2021). An *in vivo* model has shown that exosomes secreted in the saliva of *A. maculatum* and *I. scapularis* at the bite site modulate the production of cytokines or chemokines such as IL-8 and/or C-X-C motif chemokine ligand 12, which are responsible for controlling tissue injury, healing, and the wound-repair process for a successful blood-feeding mechanism (Zhou et al., 2020). This may allow and ease salivary exosomal secretions in tick feeding; understanding of its molecular mechanisms in host immunomodulation is in its infancy and will require experimental evidence to uncover its precise functional role. Future literature on ncRNAs and developing projects on the identification of the putative roles tick sialome-derived ncRNAs could be a sophisticated/possible way to describe the inhibitory activities in tick–host crosstalk.

### MicroRNAs (miRNAs) 

MiRNAs are a class of short non-coding RNAs with a length of approximately 22 nucleotides that are responsible for regulating gene expression at the post-transcriptional level and that can bind the 3’ UTR region of target mRNA to induce post-transcriptional inhibition (Ahmad et al., 2021). A single miRNA might bind several different mRNA targets, or multiple miRNAs can target a single mRNA transcript (Hermance et al., 2019). This phenomenon impedes the identification of miRNA-mRNA target interactions for discovering the regulatory network governed by miRNAs (Riolo et al., 2021). MiRNAs control several other cellular activities—growth and metabolisms (Malik et al., 2019) and blood-feeding (Hermance et al., 2019)—and strengthen tick–host interaction (Chavez et al., 2019; Chavez et al., 2021).

Although miRNAs have been identified and secreted *via* tick-derived EVs, limited studies have been conducted on the differential expression of miRNAs, with the exception of tick species that include all developmental stages of *R. microplus* (Barrero et al., 2011), gender-based dynamics in *R. sanguineus* (Shao et al., 2015), lipopolysaccharide-induced patterns in *R. haemaphysaloides* (Wang et al., 2015), differential expression in SG of *Hae. longicornis* (Zhou et al., 2013; Malik et al., 2019) and *H. anatolicum* (Luo et al., 2021), and the saliva of *I. ricinus* (Hackenberg et al., 2017). A subsequent study has shown the existence of some significant miRNAs in the EV-like structures secreted by tick sialome (36 known and 34 novel miRNAs), which may be used in tick–host interface modulation (Nawaz et al., 2020). Various tick-specific miRNAs have been identified, and their functional characteristics have been reported. For instance, the presence of miRNAs in the saliva of *I. ricinus* and its corresponding role in miRNA-mediated host gene expression regulation presented the first evidence in the tick–host interaction (Hackenberg et al., 2017), which was followed by the identification of several other miRNAs in several other tick species, including miR-275, miR-375, and miR-184 in *Hae. longicornis*, which are involved in blood digestion and oviposition (Hao et al., 2017; Malik et al., 2019); miR-133 (downregulated) and miR-79 (upregulated) in *I. scapularis* enhance the transmission of *Anaplasma phagocytophilum* from ticks to hosts (Ramasamy et al., 2020), and iri-miR-317-3p, in *I. ricinus* acts as a putative blood-feeding regulatory mechanism (Hackenberg et al., 2017). The same tick also secretes mir-317-3p, miR-8-3p, bantam-3p, and miR-279a-3p that could be the foci of research because of their role in KEGG pathways - “gap junction pathway” and “inflammatory mediator of TRP channels regulation of the host,” - depicting their crucial role in maintaining the host’s homeostatic activities in the tick–host interaction.

Little focus has been given to the miRNA profile in the sialome of ticks that could tackle the host’s rigorous immune responses except for recently discovered saliva-mediated EVs that strongly affect the dendritic epidermal T cells in *in vitro* experimentation (Chávez et al., 2021). The prospects for better understanding vector biology require the identification of miRNAs on a large scale (Malik et al., 2019). Concerning ticks, the miRNA information is limited only to characterized sequences, localized tissues, and evolutionary linkage in only five tick species. This is further scarce by the availability of annotated miRNA catalogues for only two tick species, including *I. scapularis* and *R. microplus* (Hackenberg et al., 2017). The salivary composition of other tick genera containing non-proteinaceous components—specifically miRNA expression patterns and its effects on host defense mechanisms—requires proper experiments. The identification of miRNAs in tick sialome is another advantage to demonstrating the molecular mechanisms involved in the interaction between the tick-pathogen-host interface. Their identification may also facilitate the discovery of novel targets that could potentially control ticks and their associated pathogens. Furthermore, recent updates on EVs have shown their remarkable activities in the secretion and transfer of miRNAs, lipids, and proteins intercellularly and their disposal of unimportant cellular debris (Nawaz et al., 2020). The secretion of exosomal miRNAs was further studied and found to be dependent on a ceramide-dependent pathway (O’Brien et al., 2018). Researchers have approached the objective of miRNA secretion, but the study of the uptake mechanisms of miRNAs is still in its infancy. This phenomenon has been discussed in several research articles in which multiple mechanisms were suggested/inferred; however, strong evidence is still elusive (Chen et al., 2012; Wei et al., 2017). Tick saliva-derived miRNA requires fundamentals on the molecular mechanism of its target mRNA inhibition and functional role in the immunomodulation of the tick–host interface (Malik et al., 2019). These approaches are beneficial in the development of tick miRNA-based vaccine candidates.

## Differential Expression and Secretion of Tick Secretome

Tick sialome is comprised of a wide range of proteins that are secreted dynamically and that enhance host immunomodulation during blood-feeding (Narasimhan et al., 2020; Ribeiro and Mans, 2020). The dynamic expression of tick salivary secretome varies with a tick’s blood-feeding phases, tackling host immune responses, and pathogen transmission (tick–pathogen–host interaction) depending on the species and the life stage of the tick (Kim et al., 2016; Tirloni et al., 2017). This mechanism is called *sialome switching* (Perner et al., 2018), which has been addressed in *I. scapularis* (Lewis et al., 2015; Kim et al., 2016), *I. ricinus* (Schwarz et al., 2014), *A. americanum* (Radulović et al., 2014), *R. microplus* (Tirloni et al., 2014; Garcia et al., 2020), *R. sanguineus* (s.l.) (Tirloni et al., 2020), *O. erraticus* (Pérez-Sánchez et al., 2021; Pérez-Sánchez et al., 2022), *R. pulchellus* (Tan et al., 2015), *R. zambeziensis* (De Castro et al., 2017), and *H. dromedarii* (Bensaoud et al., 2019a). As a result, these experiments have proven the presence of several putative antigenic candidates in tick sialome-derived proteomes (Pérez-Sánchez et al., 2021) that have important biological functions, particularly host attachment, blood-feeding, and modulation of the host’s defense mechanisms (Kazimírová and Stibraniova, 2013; Šimo et al., 2017; Pérez-Sánchez et al., 2021). Several studies have observed the differential expression of some specific salivary molecules. For instance, AsKunitz, As8.9kDa, and AsBasicTail have been studied in *A. sculptum* and found to be upregulated at distinct levels (larvae and nymphs: 2.4- to 745-fold; adults: 365- to almost 20 million-fold) in early blood-feeding (Esteves et al., 2017; Costa et al., 2021). In contrast, *Hae. longicornis*-derived Hlcyst-2 has shown negligible expression in the sialome, which might not be involved in blood-feeding or the immunomodulation of the host (Zhou et al., 2006). *I. persulcatus* Ipis-1 proteins have been detected in the SGs expressed at the same level throughout all phases of feeding. However, the expression of some proteins, including HSP16, has increased from the same tick increases during engorgement (Xu et al., 2005). A homologous innexin protein was found downregulated in the salivary secretion/secretome of *A. americanum* (Aljamali et al., 2009), and *O. moubata* (Díaz-Martín et al., 2013) during blood-feeding. Female *A. variegatum* fed on goats expresses 336 proteins as compared to *D. andersoni* fed on cattle, which expressed 677 proteins (Mudenda et al., 2014) and *O. moubata*, which expressed 193 proteins (Díaz-Martín et al., 2013). A large set of salivary proteins have been observed to express in different feeding phases with known and unknown functions (Rodrigues et al., 2018). This phenomenon also indicates the existence of several unknown proteins in the salivary repertoire at tick–host interface. Remarkably, qualitative and quantitative variations in the saliva secretome during different feeding stages of *R. microplus* have been suggested to modulate the expression of proteins for successful blood-feeding, and to evade the host defense mechanism (Dai et al., 2012; Tirloni et al., 2014; Kim et al., 2016; Ribeiro and Mans, 2020). This time-dependent and dynamic expression of tick salivary proteins further indicates the participation of these molecules in different intervals of blood-feeding that are associated with feeding progression (Schwarz et al., 2013; Karim and Ribeiro, 2015; De Castro et al., 2017). Molecular mechanisms underlying the differential expression of several known and unknown salivary proteins need further investigations (Karim and Ribeiro, 2015; Sajiki et al., 2020). The rapid changes in the sialotranscriptome underlying the sialome switching of ticks may be an interesting target for the control of ticks and tick-borne pathogens.

Tick sialome also influences the acquisition, propagation, and transmission of a large array of pathogens (Pham et al., 2021). Tick-acquired pathogens exploit the activity of salivary molecules to enhance their survival and transmission (Karim and Ribeiro, 2015; Perner et al., 2016), and facilitate blood-feeding, and immune evasion at tick-host interface (Nuttall, 2019). Saliva assisted pathogens contribute to the dynamic expression of several molecules in the SGs of ticks (Kazimírová and Stibraniova, 2013). For instance, salivary Salp15 protein has been reported to selectively overexpress in the SGs of *I. scapularis* nymphs and bind a *Borrelia burgdorferi* derived OspC, a spirochete surface protein (Ramamoorthi et al., 2005). This pathogen enhances the expression of tHRF in *I. scapularis* (Dai et al., 2010), and transcription of TSLPI which binds the active sites of C-type mannose binding lectin of the host complement pathway (Dulipati et al., 2020). *B. afzelii* increases the expression of Salp15 in *I. ricinus* (Hovius et al., 2008b). *Anaplasma phagocytophilum* enhances the expression of Salp16 in the *I. scapularis* (Sukumaran et al., 2006). Among others, upregulation of Salp11, metis-1, prolyl 4-hydroxylase and cement proteins have been observed in ticks infected with *A. phagocytophilum* and *B. burgdorferi* (Cotté et al., 2014). Substantial up-regulate of homolog of histone deacetylase 1 protein in *I. scapularis* has been observed in SGs against *A. phagocytophilum* infection (Cabezas-Cruz et al., 2016). *Rickettsia parkeri* dynamically interact with *A. maculatum* symbionts and upregulate tick selenoproteins (Budachetri et al., 2018). Recent investigation on tick-borne Powassan virus described its ability to alter the expression of miRNAs in the SGs of *I. scapularis* (Hermance et al., 2019). Sialome switching could also be triggered by the epigenetic regulation in ticks; mediated by histone modification and chromatin remodeling (Adamson et al., 2013; Kotsyfakis et al., 2015; Cabezas-Cruz et al., 2016), stressor signals, internal clock of tick species, transcription factors, response to a pathogenic infection, and rigorous innate and acquired immune responses of the host, or contribution of both tick-host factors (Karim and Ribeiro, 2015; Perner et al., 2018). Moreover, the composition of saliva varies, and tick express specific proteins when they are exposed to different host species (Tirloni et al., 2017; Narasimhan et al., 2019). Several factors are involved in the induction of sialome switching at tick-host interface however, the molecular nature of sialome switching and its effects on the chain of biological processes are mostly remained unknown. Therefore, further research is essential to assess the molecular mechanisms of sialome switching during blood-feeding phases, and sialome manipulation by saliva-assisted pathogens in different tick species that might provide important information about tick salivary molecules essential for the control of ticks and tick-borne pathogens.

## Role of Omics in Salivary Secretome

Knowledge of the composition and variations in the expression of tick saliva proteins is essential for understanding the tick feeding process and host immunomodulation mechanisms (Tirloni et al., 2020). Understanding the complexity of tick saliva proteins, redundant activities, putative roles, and differential gene expressions during blood-feeding has increased over the past decades as sophisticated approaches have been opted for at cellular, molecular, genomic, functional genomic, and proteomic levels (Wikel, 2018). Computational methods that enable the exploration of the immunomodulatory activities of a large set of tick salivary molecules have been identified; however, most remain to be tested in clinical trials. Currently, transcriptomics, proteomics, large-scale DNA sequencing, and bioinformatics analyses have been used to identify genes and proteins essential for resolving the complexity in the tick–host critical facets that facilitate the salivary composition and molecular dynamics throughout tick blood-feeding (Martins et al., 2020; Tirloni et al., 2020; Boulanger and Wikel, 2021). The sialoproteomes and sialotranscriptomes of several tick species have been annotated, along with their putative roles in the modulation and inhibition of host hemostatic, inflammatory, and immunity processes, in various reviews (Francischetti et al., 2008; Chmelař et al., 2012; Kotál et al., 2015; Martins et al., 2021; Pérez-Sánchez et al., 2021). The effective control of ticks and tick-borne pathogens is a long-standing and worldwide challenge (Willadsen, 2004; De la Fuente and Contreras, 2015; De la Fuente et al., 2016; De la Fuente et al., 2017; Van Oosterwijk and Wikel, 2021). Several attempts have been made to identify effective antigens in the tick sialome that could deter the blood-feeding and block the pathogen transmission (Willadsen, 2004; Ramamoorthi et al., 2005; Dai et al., 2010; Rego et al., 2019). Although commercial and experimental vaccine formulations have elicited partial protective immune responses against ticks, until now, there are no vaccine that provides adequate levels of protection against tick infestation. The use of anti-tick vaccines rises a potential alternative control method, making tick vaccine development economically essential (Parizi et al., 2012; De la Fuente and Contreras, 2015; De la Fuente et al., 2016; De la Fuente et al., 2017). Recent advances in the transcriptomic and proteomic approaches through next-generation sequencing (NGS) have increasingly resolved the complexities of tick salivary compositions during blood-feeding in different developmental stages (Bensaoud et al., 2019a). Several factors have hampered the successful development of anti-tick vaccine including the lack of understanding of the complex mechanism of tick-pathogen-host interaction, salivary molecule’s assisted microbial diversity, selection of suitable protective antigens that can induce considerable protection and potentially target a broad range of ticks and tick-borne pathogens (Rego et al., 2019; Van Oosterwijk and Wikel, 2021). Possibly, the main difficulty to obtain an adequate vaccine against ticks is the lack of knowledge about the mechanisms involved in host immune responses that induces tick rejection. Therefore, strategies should be designed to decipher the genetic basis of tick-host-pathogen interactions which are the crucial in the development of salivary derived anti-tick vaccine.

NGS further promotes the characterization of several tick sialome sequences and has opened new horizons for the discovery of mechanistic studies regarding the tick–host interface (Martins et al., 2020; Oleaga et al., 2021a). Advancements in next-generation RNA sequencing and a label-free quantitative proteomics have been used to demonstrate the quantitative gene expression in the midgut and SGs of ticks during host attachment (Schwarz et al., 2014). Massive analysis of cDNA Ends sequencing approaches in combination with RNA sequencing is useful for determining up and downstream regulation expression of tick salivary proteins during tick feeding (Rego et al., 2019; Trentelman et al., 2020). A quantitative isobaric tag for relative and absolute quantitation proteomics strategy has been developed for investigating differential protein expression in different feeding stages of tick SGs (Ren et al., 2019). Small quantities of proteins can be targeted by employing several techniques such as peptide mass fingerprinting by matrix-assisted laser desorption/ionization-mass spectrometry and shotgun proteomics by precursor ion detection and product ion detection (Rego et al., 2019). In addition, the protein half-life in the circulation can be extended by using proline/alanine-rich sequence protein conjugation with the polymeric sequence (Pro, Ala, and Ser) (Schlapschy et al., 2013; Chmelař et al., 2019).

Several complexities about the immunological interaction at tick-host interface have been increasingly resolved using genome arrays (Van der Heijden et al., 2005; Heinze et al., 2012). A complete sequence dataset of ticks is required to unravel the molecular mechanisms behind the differential expression and regulation of tick salivary proteins during feeding phases (De Castro et al., 2017). The genomic and transcriptomic data of ticks, tick-borne pathogens, and pathogen’s infected tick can provide an aid in selection and characterization of the novel therapeutic targets and vaccine candidates (Sette and Rappuoli, 2010; Antunes et al., 2012; Cramaro et al., 2015; De la Fuente and Contreras, 2015; Blecha et al., 2018; Davies et al., 2019; Ali et al., 2020a; Ali et al., 2022; Jia et al., 2020). In recent years, a number of genomic and sialotranscriptome sequences have been annotated (Hill and Wikel, 2005; Guerrero et al., 2006; Wang et al., 2007; Aljamali et al., 2009; Anatriello et al., 2010; Guerrero et al., 2010; Francischetti et al., 2011; Karim et al., 2011; Ribeiro et al., 2012; Schwarz et al., 2013; Garcia et al., 2014; Mudenda et al., 2014; Schwarz et al., 2014; Cramaro et al., 2015; Karim and Ribeiro, 2015; Xu et al., 2015; Yu et al., 2015; De Castro et al., 2016; De la Fuente et al., 2016; Gulia-Nuss et al., 2016; Barrero et al., 2017; De la Fuente et al., 2017; Maruyama et al., 2017; Ribeiro et al., 2017; Antunes et al., 2018; Miller et al., 2018; Nuss et al., 2018; Garcia et al., 2020; Jia et al., 2020; Couto et al., 2021; Oleaga et al., 2021b). Recently published annotated genome sequences of different tick species and their associated pathogens provide a valuable resource to understand blood-feeding mechanisms, tick-host-pathogen interactions, and genetic basis as tool for the control of ticks and tick-borne pathogens (Jia et al., 2020). The availability of such information affords unprecedented insight into the complex mechanisms in tick sialome and the temporal expression of several secretory protein families. Therefore, fundamental knowledge about tick genome is essential in the exploration of new horizons about tick biology, tick-host-pathogen interactions, and control strategies.

Advances in the field of computational biology have substantially improved several PIs and their targeted proteases by using 3D structural information that could be useful in permitting receptor-based design (Jmel et al., 2021). Recently discovered ncRNAs in tick saliva have been proposed to be prompted by the immune modulators of the vertebrate host (Chen et al., 2019). Many ncRNA sequences have been characterized as playing some role in the modulation of host gene expression through binding miRNAs to the host regulatory mRNAs (mRNAs) (Zhou et al., 2013; Bensaoud et al., 2019b; Malik et al., 2019; Aounallah et al., 2020; Nawaz et al., 2020). These may be concerned with the regulation of tick development or blood-feeding that has been studied in several tick species and described as gene regulators (Boulanger and Wikel, 2021). Recent updates on tick salivary miRNAs have raised growing concern regarding their redundant functions in gene expression regulation, targeting mRNAs, and tick–host interaction (Zhou et al., 2013). Studies are needed to predict miRNA molecular targets in the vertebrate host that may assist future tick-control methods (Hackenberg et al., 2017). Complexity in tick–host molecular interactions is also required for in silico screening of tick sialomes with important immunogenic properties and their directed changes in the host defense mechanism, followed by wet-lab verification (Bhowmick and Han, 2020; Ali et al., 2020a; Ali et al., 2020b). Therefore, the implementation of bioinformatics tools and bioassays to monitor the amounts of target mRNA (Ekimler and Sahin, 2014), a computational approach such as algorithms scripted from mRNA sequences and/or based on the miRNA-mRNA interactions, machine learning that describes the statistical inference (Riolo et al., 2021), and small RNA high-throughput sequencing (Hermance et al., 2019) are necessary to predict miRNA targets and rule out the molecular mechanism for maintaining the integrity of the tick–host interface. The biochemical characterization of tick salivary components in the tick–host interface is central to understanding the genes, proteins, motif, or residues and subsequently elucidate their functional mechanisms, role in the activation or inhibition of specific enzymes or receptors, blood coagulation, and platelet aggregation (Valenzuela, 2004). Biochemical analyses are useful in the identification of miRNA targets in a way more sophisticated than genetic methods (Ekimler and Sahin, 2014). Over the last two decades, tick salivary proteome has been studied, revealing that the protein profile of tick sialome is complex (Narasimhan et al., 2007; Francischetti et al., 2009; Kim et al., 2016; Perner et al., 2018; Ali et al., 2020b). Exploring the structure and functional mechanisms of tick salivary secretome and host target molecules can be used to develop synthetic peptides (Maritz-Olivier et al., 2007; Koh et al., 2018). Some examples have included the mapping of thrombin exosites by ornithodorin (Van der Locht et al., 1996), Ixolaris-derived prothrombinase complex formation (Monteiro et al., 2005), and molecular mechanisms that maintain the fV and its conversion to fVa using recombinant TIXC-5 (Aleman and Wolberg, 2013; Schuijt et al., 2013). However, novel control methods are restricted due to the lack of understanding the fundamentals of tick biology and mechanisms underlying the tick–host molecular interface (Slunge, 2015).

The omic era has advanced our understanding of the functional origin and evolution of the complex tick and host proteins that interplay during tick–host crosstalk (Vayssier-Taussat et al., 2015). The basic understanding of tick biology; the molecular interface between tick and host; the differential secretion of tick salivary secretome in the tick’s developmental stage, gender, feeding time, and behavior; and the upregulated or downregulated host genes during tick infestation have been mostly disclosed by NGS (Martins et al., 2020). Exploiting novel downregulated tick proteins and upregulated host proteins during tick feeding may be vital to hematophagy and, consequently, to suitable candidate antigens (Tabor et al., 2017). The advanced strategies increase our understanding of specific and unique tick genes and proteins, along with the annotation and classification of identified sequences (Tirloni et al., 2020). Transcriptomes and proteomes of tick sialomes have been annotated in vaccinomic pipelines for the selection and characterization of candidate protective anti-tick vaccines (Pérez-Sánchez et al., 2021), supported by techniques in the development of new proteins (Tripathi and Shrivastava, 2019), including immuno-proteome methods (Narasimhan et al., 2007), yeast surface display (Rego et al., 2019), 1D-PAGE/tryptic digestion/RPLC/MS/MS (Francischetti et al., 2008), RNA interference (RNAi), and CRISPR-Cas9 technologies (Barnard et al., 2012; Sharma et al., 2020). Broadly speaking, a number of experimental methods, including RNAi, expression library immunization and sequences tags, interactomics, proteomics, and transcriptomics, are involved in the identification of putative vaccine candidates through which a cocktail of vaccines can be selected that could increase the potential synergistic effects against tick infestation (Kocan et al., 2003; Artigas-Jerónimo et al., 2018).

Omics’ explosion of data further improves the reverse vaccinology strategies applied as an alternative for the detection of novel candidates for next-generation diagnostics and vaccines (De la Fuente et al., 2020; Couto et al., 2021). These approaches have revolutionized screening for novel natural substances. Furthermore, computational biology approaches have been introduced that assembled previously published data in a single platform such as miRNA sequences and annotations in the MicroRNA Sequence Database (miRBase) (Mans, 2019; Perez-Riverol et al., 2019), raw proteomic data in the PRoteomics IDEntifications Database (Mans, 2020), computationally assembled transcript sequences in the Transcriptome Shotgun Assembly, annotated tick sialotranscriptomes in TickSialoFam (Ribeiro and Mans, 2020), and annotated genomic assembly in VectorBase (Giraldo-Calderón et al., 2015). These databases are accessible and updated by adding new sequences and annotation details that facilitate the scientific community (Ribeiro and Mans, 2020; Marceca et al., 2021). Several research studies have provided their supplementary data comprised of putative salivary molecules derived from various tick species, which provides a valuable reference database for the current ongoing transcriptomic and proteomic studies (Oleaga et al., 2021b) and assists the process of functionally identifying unique transcripts and their associated proteins. The generation of these large data sets is the foundation for understanding the global perspective of tick–host interaction that contributes to the initiation of studies linking specifical immunomodulatory events with specific tick-derived molecules (Wikel, 2018) and high-throughput screening of compound libraries for target-oriented molecular models (Chmelař et al., 2019). In the best-case scenario, intense research efforts have been made in the last few years to achieve long-term goals for the identification of tick-derived antigens that may be useful in blocking successful feeding and pathogen transmission and that could be exploited for an anti-tick vaccine (Parizi et al., 2015; Martins et al., 2020; Pérez-Sánchez et al., 2021; Sajid et al., 2021). To date, a limited number of sialome-derived proteins have been functionally characterized in different ticks; however, the sialome repertoire and biological activities, along with post-translational gene modification by proteomic analysis, are far beyond our understanding. Subsequently, the systematic classification of the tick–host molecular interaction has remained incomplete due to less coverage of the transcriptome and proteome spaces. Comprehensive understanding may require improvement in the current technology; however, taking advantage of the recent omic era, the rich cocktail of salivary molecules in various tick species can be determined, along with the remarkable molecular mechanisms and affinity in the tick–host interface, perhaps providing a pipeline to successful functional descriptions of these molecules.

## Conclusion

Updated knowledge about understanding the tick–host molecular interface is addressed, which may assist strategies to successfully deter tick blood-feeding and direct the development of effective anti-tick control. The molecular interaction between tick salivary molecules and host components throughout tick blood-feeding has been briefly discussed for understanding the roles of these molecules in the modulation of host responses. Based on growing knowledge of the complexity of tick–host molecular interaction, further experiments using functional genomics such as the CRISPR system may shape the components involved in crosstalk and control approaches. In addition, the specific roles and activities of molecules derived from ticks and their diverse means of action against host defense mechanisms in ticks remain to be investigated. Specifically, extensive studies on the molecular and cellular level of tick sialome–derived miRNAs are required to decipher their putative roles in tick–host interactions and, in turn, enhance discoveries of vaccine candidates for clinical trials. These gaps in the existing knowledge could be unraveled by introducing technological advancements that may lead to unprecedented information on the tick–host interaction and effective control strategies.

## Author Contributions

AAli, IZ, and HZ searched and collected the literature and wrote the manuscript. AAli, MMA, AAlo, FA, MA, CT, IV, and TT supervised the overall investigations and helps in the manuscript editing. All authors contributed to the article and approved the submitted version.

## Funding

This work was supported by the JST Adaptable and Seamless Technology Transfer Program through Target-driven R&D (A-STEP) Grant Number JPMJTM20SV and Takeda Science Foundation.

## Conflict of Interest

The authors declare that the research was conducted in the absence of any commercial or financial relationships that could be construed as a potential conflict of interest.

## Publisher’s Note

All claims expressed in this article are solely those of the authors and do not necessarily represent those of their affiliated organizations, or those of the publisher, the editors and the reviewers. Any product that may be evaluated in this article, or claim that may be made by its manufacturer, is not guaranteed or endorsed by the publisher.
